# Construction of a Prognostic Model in Lung Adenocarcinoma Based on Ferroptosis-Related Genes

**DOI:** 10.3389/fgene.2021.739520

**Published:** 2021-09-22

**Authors:** Min Liang, Mafeng Chen, Yinghua Zhong, Shivank Singh, Shantanu Singh

**Affiliations:** ^1^ Department of Respiratory and Critical Care Medicine, Maoming People’s Hospital, Maoming, China; ^2^ Department of Otolaryngology, Maoming People’s Hospital, Maoming, China; ^3^ Department of Pediatrics, Fogang County Hospital of Traditional Chinese Medicine, Qingyuan, China; ^4^ Southern Medical University, Guangzhou, China; ^5^ Division of Pulmonary, Critical Care and Sleep Medicine, Marshall University, Huntington, WV, United States

**Keywords:** lung adenocarcinoma, ferroptosis, gene, prognosis, prognostic

## Abstract

**Background:** Lung adenocarcinoma is one of the most common malignant tumors of the respiratory system, ranking first in morbidity and mortality among all cancers. This study aims to establish a ferroptosis-related gene-based prognostic model to investigate the potential prognosis of lung adenocarcinoma.

**Methods:** We obtained gene expression data with matching clinical data of lung adenocarcinoma from the The Cancer Genome Atlas (TCGA) and Gene Expression Omnibus (GEO) databases. The ferroptosis-related genes (FRGs) were downloaded from three subgroups in the ferroptosis database. Using gene expression differential analysis, univariate Cox regression, and LASSO regression analysis, seven FRGs with prognostic significance were identified. The result of multivariate Cox analysis was utilized to calculate regression coefficients and establish a risk-score formula that divided patients with lung adenocarcinoma into high-risk and low-risk groups. The TCGA results were validated using GEO data sets. Then we observed that patients divided in the low-risk group lived longer than the overall survival (OS) of the other. Then we developed a novel nomogram including age, gender, clinical stage, TNM stage, and risk score.

**Results:** The areas under the curves (AUCs) for 3- and 5-years OS predicted by the model were 0.823 and 0.852, respectively. Calibration plots and decision curve analysis also confirmed the excellent predictive performance of the model. Subsequently, gene function enrichment analysis revealed that the identified FRGs are important in DNA replication, cell cycle regulation, cell adhesion, chromosomal mutation, oxidative phosphorylation, P53 signaling pathway, and proteasome processes.

**Conclusions:** Our results verified the prognostic significance of FRGs in patients with lung adenocarcinoma, which may regulate tumor progression in a variety of pathways.

## Background

Lung cancer is the most commonly diagnosed malignant tumor worldwide, whose morbidity and mortality rate rank first among all cancers, and the severity is increasing year by year, posing a great threat to human health (Sung et al., 2021). According to pathological classification, the disease can be categorized as small cell lung adenocarcinoma and non-small cell lung cancer (NSCLC), among which, the latter accounts for about 2/3 cases (Rodríguez-Martínez et al., 2018). NSCLC can be divided into three types, including lung adenocarcinoma, squamous cell lung cancer, and non-small cell lung cancer, of which lung adenocarcinoma accounts for about 40% of cases (Bender, 2014). Epidemiological data reveal that the 5-years overall survival rate of lung cancer in all stages is as low as 15.9% (Ettinger et al., 2013). Therefore, it is of great importance to find biomarkers closely associated with the prognostic outcomes of lung cancer, especially lung adenocarcinoma, as well as to evaluate the prognosis outcome of patients with squamous cell lung cancer through these markers, which can improve the prognosis and formulate individualized diagnosis and treatment (Santarpia et al., 2020).

Ferroptosis, a relatively novel kind of cell death discovered recently, is involved in the pathophysiological process of many diseases including tumors (Wu et al., 2019), and it is different from apoptosis, necrosis, and autophagy due to a feature: being iron-dependent. It is caused by the accumulation of toxic lipid reactive oxygen species and the consumption of polyunsaturated fatty acids (Li et al., 2020). Polyunsaturated fatty acid is an important substrate in ferroptosis, and the C–H bond in the diallyl group of polyunsaturated fatty acid is easily attacked by oxidation. Compared with normal cells, cancer cells have the phenomenon of iron ion aggregation, and the regulation of ferroptosis from the perspective of iron homeostasis can effectively kill tumor cells (Lei et al., 2021). In recent years, for the treatment of advanced tumors, especially drug-resistant tumors, inducing the death of cancer cells through ferroptosis has become a very promising option (Xu et al., 2021). In addition to various induction molecules, many genes can also be markers of ferroptosis (Chen et al., 2021a). At present, ferroptosis-related genes have shown good predictive performance in not a few tumors, including glioma (Zhuo et al., 2020), liver cancer (Tang et al., 2020), pancreatic cancer (Jiang et al., 2021a), gastroenteric tumor (Angius et al., 2019), urologic neoplasms (Liu et al., 2021), and thyroid cancer (Ge et al., 2021). However, the relationship between FRGs and prognosis and the outcome of patients with lung adenocarcinoma has not been reported in depth.

Therefore, our study aims to explore the role of FRGs to predict outcome, and on the basis of which, establish a prognostic model to assess the prognostic outcome of patients with lung adenocarcinoma. Based on the differentially expressed genes (DEGs) related to ferroptosis, a prognostic model was constructed according to the training set, and the predictive power of the model was verified in the validation set. Finally, we proceeded with a functional enrichment analysis to investigate the biological mechanism of FRGs in lung adenocarcinoma.

In this study, a prognostic model consisting of seven genes associated with ferroptosis was established with excellent predictive power. Enrichment analysis showed that these genes were associated with the development of lung adenocarcinoma.

## Methods

### Resources and Pre-processing

The gene expression data and related clinical information of lung tumors were extracted from the The Cancer Genome Atlas (TCGA) database (https://genome.nih.gov/). Ferroptosis-related gene sets were extracted from three subgroups in the ferroptosis database (http://www.zhounan.org/ferrdb/). Edge R package from R was used to normalize the entire data set, set |log2FC| > 0.5 and false discovery rate (FDR) < 0.05 as the threshold to construct a volcano map, to further obtain differentially expressed ferroptosis-related genes.

### Construction of the Prognostic Model

According to downloaded clinical data of lung adenocarcinoma cases from TCGA, patients with an adequate follow-up time (>30 days) were screened and divided into a training set and an internal validation set at a ratio of 2:1. The sets were divided to construct a prognostic model and verify the model, respectively. In addition, two Gene Expression Omnibus (GEO) (https://www.ncbi.nlm.nih.gov/geo/) data sets are applied for external validation of the model. Based on the training set, we performed univariate Cox regression analysis on FRGs and survival data and set *p* < 0.05 to identify differential FRGs related to prognosis, and LASSO regression was performed to further screen the genes. After obtaining seven target genes, a multi-factor stepwise Cox regression was performed to analyze their respective coefficients (βi). Finally, a risk-scoring formula consisting of βi and gene expression level (Expi) was constructed as follow:

$$\text{Risk score}=\overset{\text{i}}{\underset{\text{i}=1}{\sum }}\left({\text{βi*Exp}}_{\text{i}}\right)$$

According to the model, the risk score of invidual could be acquired. In addition, the median of the risk score was set as a critical value, and all patients included were divided into high- and low-risk groups. To reveal the prognostic outcome difference between the groups Kaplan–Meier survival curve was used. Then, the above results were verified by the validation set. To further assess the ability to predict, we conducted a subgroup analysis to compare the OS between the two groups.

### Construction of the Nomogram

We incorporated clinical features including risk score, clinical and TNM stage, age, and gender into the final model to establish a novel nomogram to predict the OS of patients individually. In addition, AUC was obtained through a receiver operating characteristic (ROC) curve to assess the accuracy of the nomogram. Subsequently, we used the calibration chart and decision curve analysis to verify the predictive ability of the model (Ge et al., 2021). The validation set was used to verify the results obtained finally.

### Gene Set Enrichment Analysis

The reference set (2. cp.kegg.v6.2. symbols.gmt, c5. all. v6.2. symbols.gmt) was downloaded from the Molecular Signatures Database (http://www.gsea-msigdb.org/gsea/msigdb/index.jsp), and the number of random combinations was set as 1,000 according to the default weighted enrichment method (NPM *p* < 0.05, FDR *p* < 0.05). The study conducted gene ontology (GO) and Kyoto Encyclopedia of Genes and Genomics (KEGG) analysis on the DEGs in the groups and deduced their functions by analyzing gene sets. Therefore, this way it can be used to clarify the question on whether the gene set shows a statistically significant difference between the two biological states. The study explored whether the DEGs between the two groups are enriched during the disease progression as well.

### Statistical Analysis

All statistical analyses were conducted using R version 4.1.0 (package: limma, pheatmap, survival, glmnet, survminer, survivalROC, rms, and timeROC). Univariate and multivariate Cox regression were used to analyze the correlation between clinical features, risk scores, and the OS of patients. The ROC curve, C-index, the calibration curve, and DCA curve were used to assess the predictive power of the model. Two-tailed *p* < 0.05 was considered statistically significant.

## Results

### Extraction of Ferroptosis-Related Genes in Lung Adenocarcinoma

**FIGURE 1:**
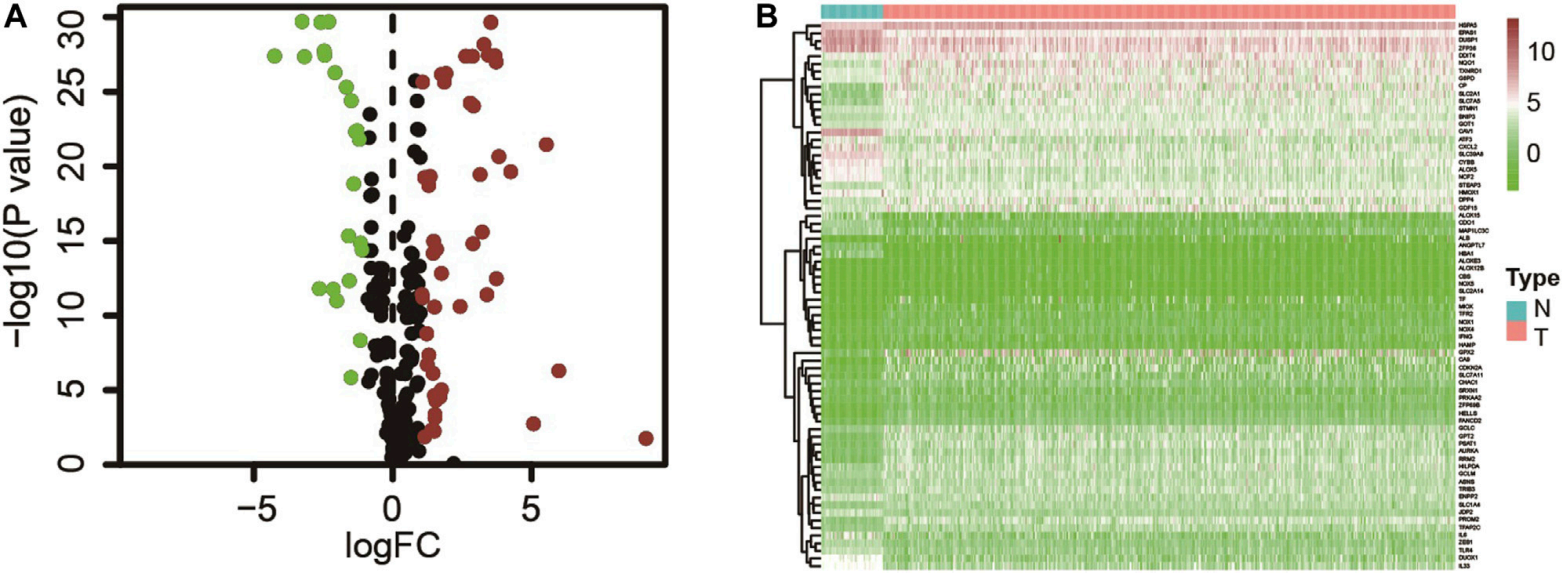
**(A)** Ferroptosis-related genes differentially expressed in tumor and adjacent tissues; **(B)** Expression of ferroptosis-related genes in tumors and adjacent tissues.

The gene expression result with matching clinical data of lung adenocarcinoma (497 tumor tissues and 54 paracancerous tissues) were extracted from the TCGA database, and the ferroptosis-related gene set (259 genes) was obtained from the ferroptosis database. Expression matrices of all ferroptosis-related genes were extracted from the TCGA dataset and differential expression analysis was performed (Supplementary Table S1). Seventy-two differential ferroptosis-related genes in lung adenocarcinoma tissues and adjacent tissues were screened out, of which 49 were upregulated, and 23 were downregulated (Figure 1 and Supplementary Table S2).

### Construction of the Prognostic Model

**FIGURE 2:**
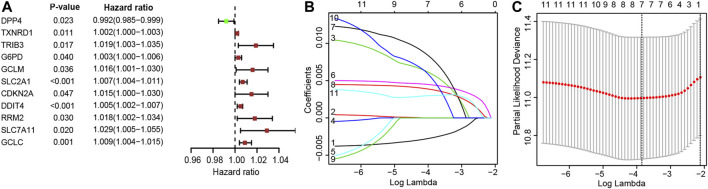
**(A)** 11 Ferroptosis-related genes significantly associated with overall survival (OS) in lung adenocarcinoma; **(B)** Adjustment parameters in LASSO regression model; **(C)** LASSO coefficient spectrum of ferroptosis-related genes.

**FIGURE 3:**
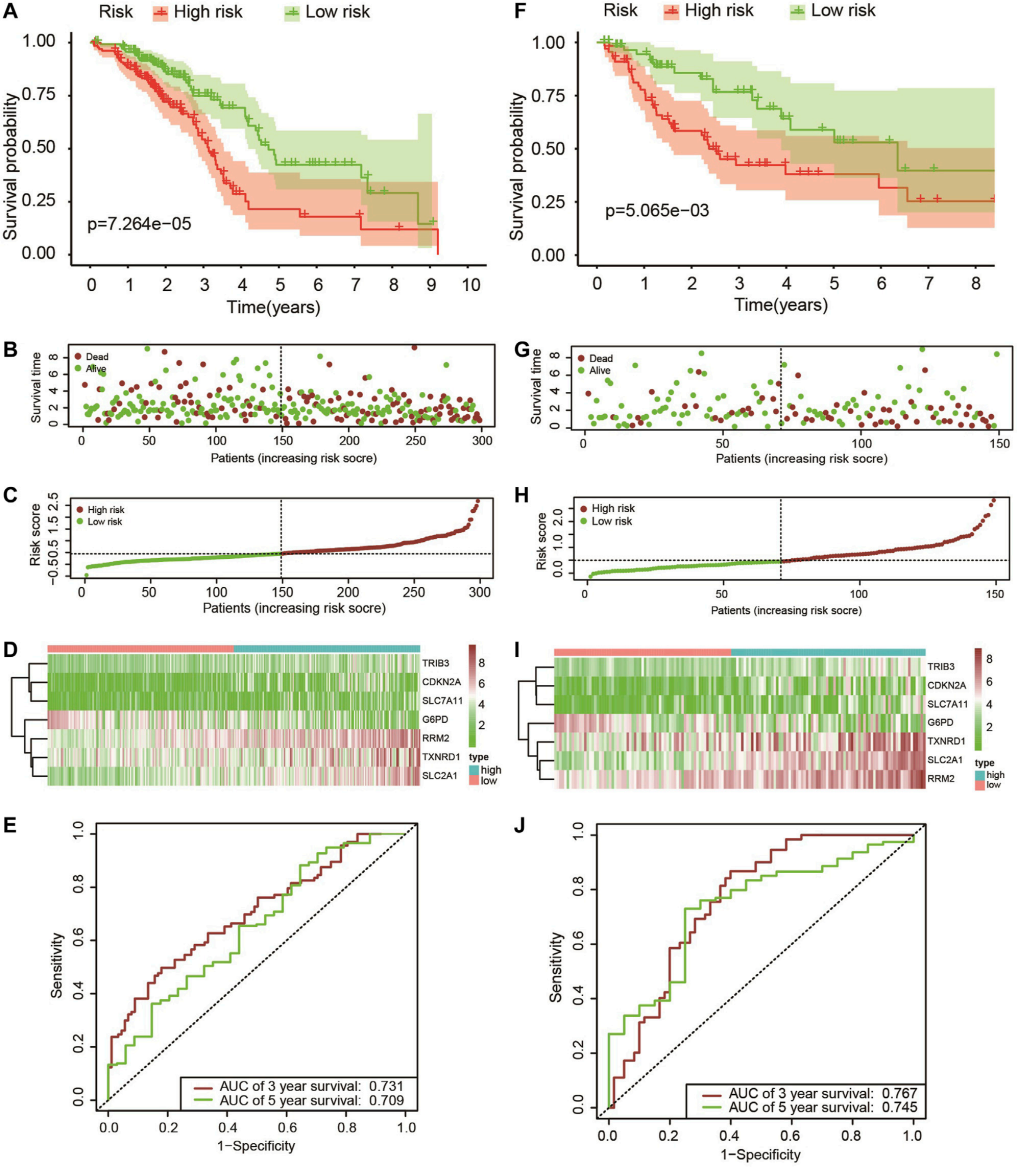
(**A–F**) Kaplan–Meier survival analysis of patients with lung adenocarcinoma in high- and low-risk groups; (**B**,**G**) The overall survival rate and status of patients with lung adenocarcinoma; (**C**,**H**) The distribution of risk scores; (**D**,**I**) The expression level of these seven ferroptosis-related genes in the low-risk group and the high-risk group, the cool color represents low expression, while the warm color represents high expression; (**E**,**J**) 3-years and 5-years ROC curve analysis of the prognostic model.

**FIGURE 4:**
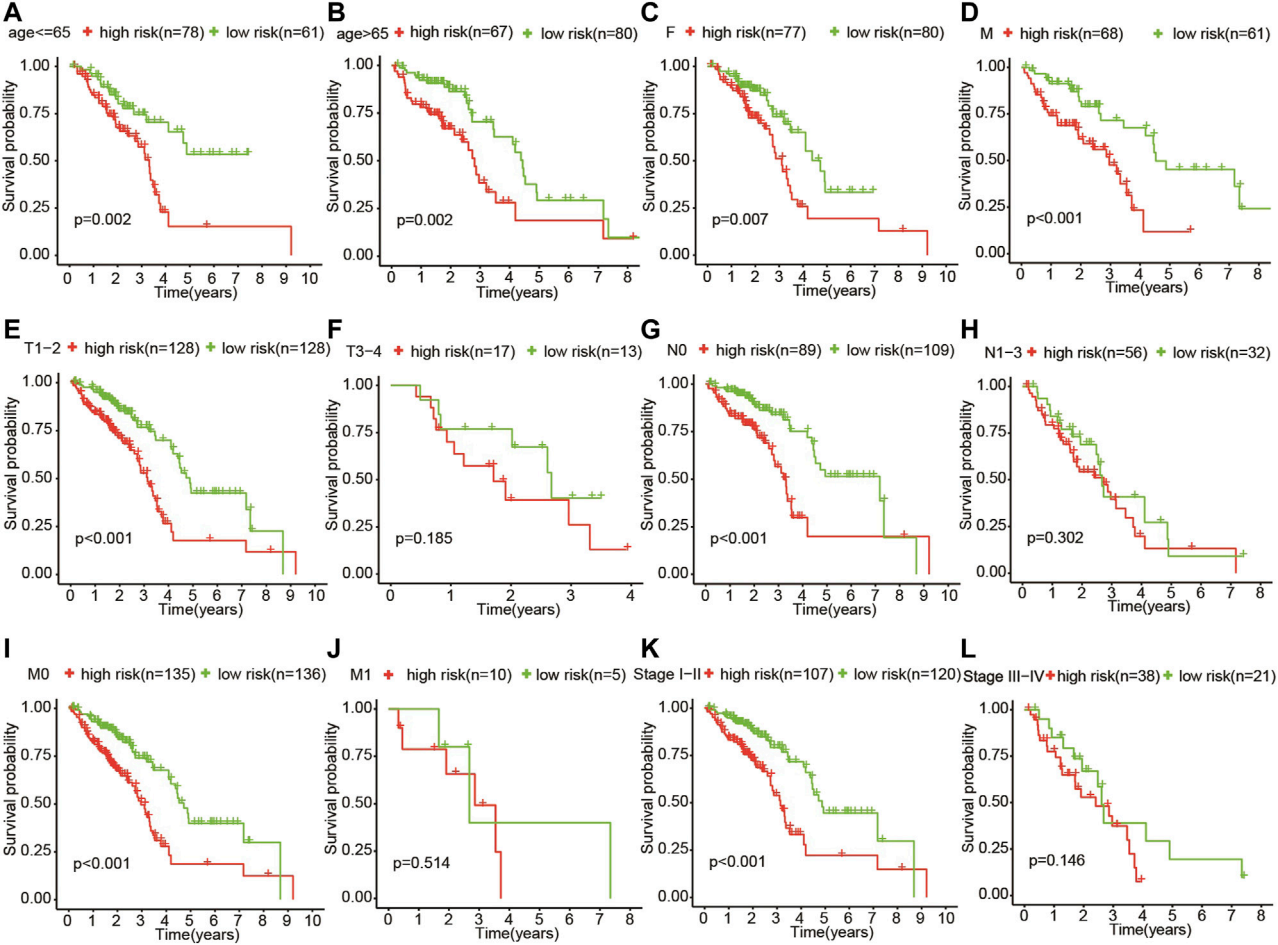
Subgroup analysis showed that according to age (**A**,**B**), gender (**C**,**D**), TNM stage (**E–K**), and clinical stage (**L**,**M**), the OS of patients in the high-risk group was more unfavorable than that in the other group.

The patients who met an adequate follow-up time (>30 days) were divided into training and validation sets at a ratio of 2 to 1. In the training set, we performed univariate Cox regression on FRGs and corresponding clinical survival data, and initially screened 11 FRGs related to prognosis (Figure 2A). Seven target genes were further screened by LASSO regression analysis, namely TXNRD1, TRIB3, SLC2A1, CDKN2A, RRM2, SLC7A11, and G6PD (Figures 2B,C). The obtained target genes were adapted to calculate the risk score of the individual through the Cox proportional hazard regression model (Supplementary Table S3). The median of the risk score was set as a cut-off value, on which basis we divided patients into two groups (Figure 3). The Kaplan–Meier curve showed that the OS of the high-risk group was worse than the other group (Figure 3A). The risk curves and scatter plots can reveal the risk score and survival status of each patient. As shown in Figures 3B,C, the mortality and risk coefficient of the high-risk group were significantly higher than that of the low-risk group. Figure 3D displays the expression profile of these seven genes. The ROC curve analysis of the 3- and 5-years OS yielded AUCs of 0.731 and 0.709, respectively (Figure 3E). Similar results were observed using the same process in the internal validation set (Figures 3F–J) and external validation (GSE37745 and GSE68465) (Supplementary Figure S1). Subgroup analysis showed that according to age, gender, TNM stage, or clinical stage, the prognosis of patients in the low-risk group are more favorable (Figure 4).

### Construction of the Nomogram

**FIGURE 5:**
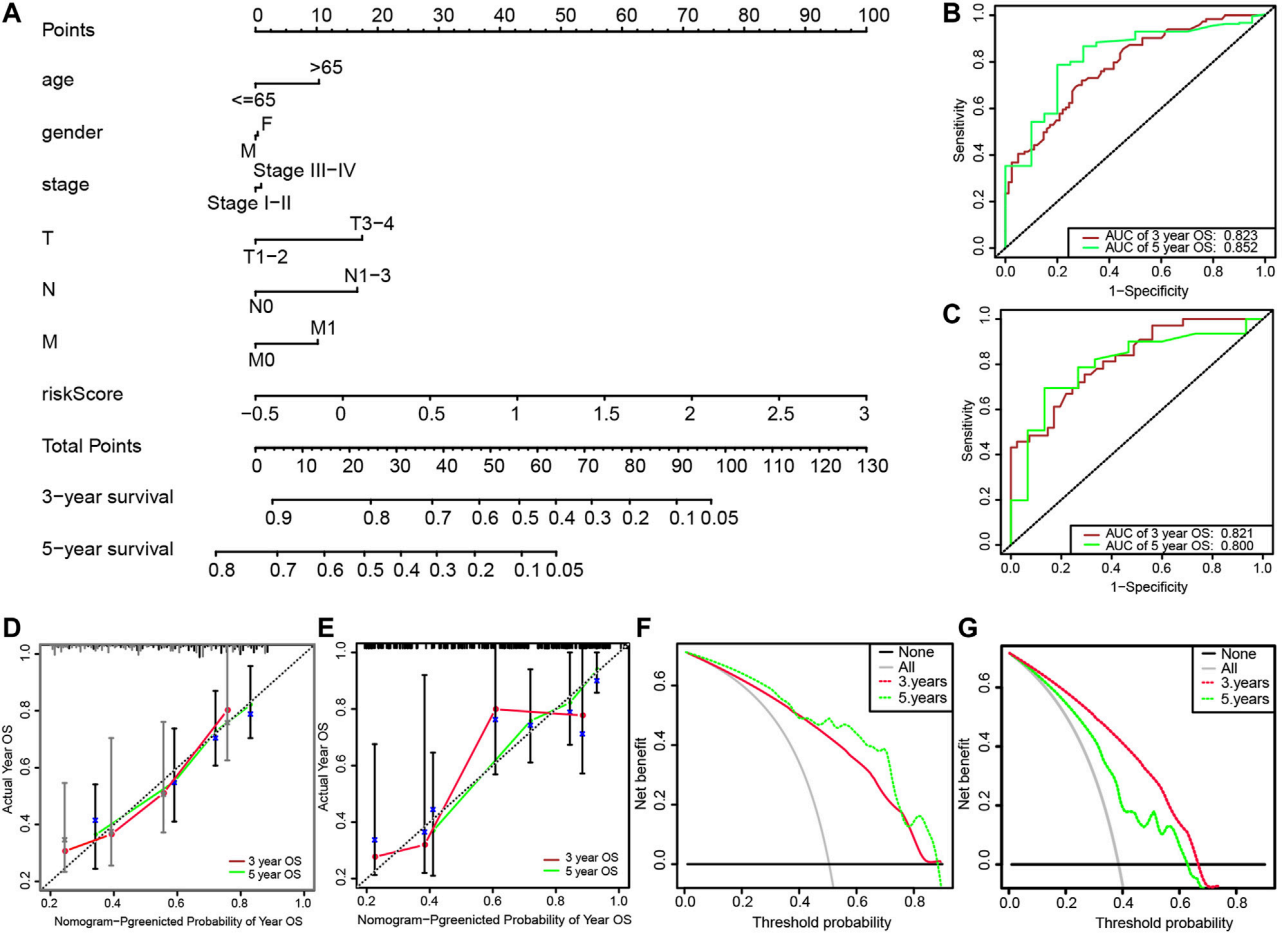
(**A**) Nomogram for predicting 3-years and 5-years survival rates of patients with lung adenocarcinoma; (**B**) ROC curve analysis for predicting 3-years and 5-years OS through nomogram in the training set; (**C**) ROC curve analysis for predicting 3-years and 5-years OS through the nomogram in the validation set; (**D**) Calibration of 3-years survival rate based on the nomogram; (**E**) Calibration of 5-years survival rate based on the nomogram; (**F**) Decision curve analysis of 3-years survival rate based on the nomogram; (**G**) Decision curve analysis of 5-years survival rate based on the nomogram.

We then developed a nomogram, including age, gender, clinical stage, TNM stage, and risk score, as shown in Figure 5A. The ROC curve analysis of the 3- and 5-years OS of the prognostic model yielded AUCs of 0.823 and 0.852, respectively (Figure 5B). The established calibration chart and decision curve analysis show that the nomogram has a favorable predictive effect (Figures 5D–G). In the validation set, the 3- and 5-years AUCs obtained by analyzing the ROC curve of the novel prediction model were 0.821 and 0.800, respectively, as shown in Figure 5C.

### Gene Set Enrichment Analysis

**FIGURE 6:**
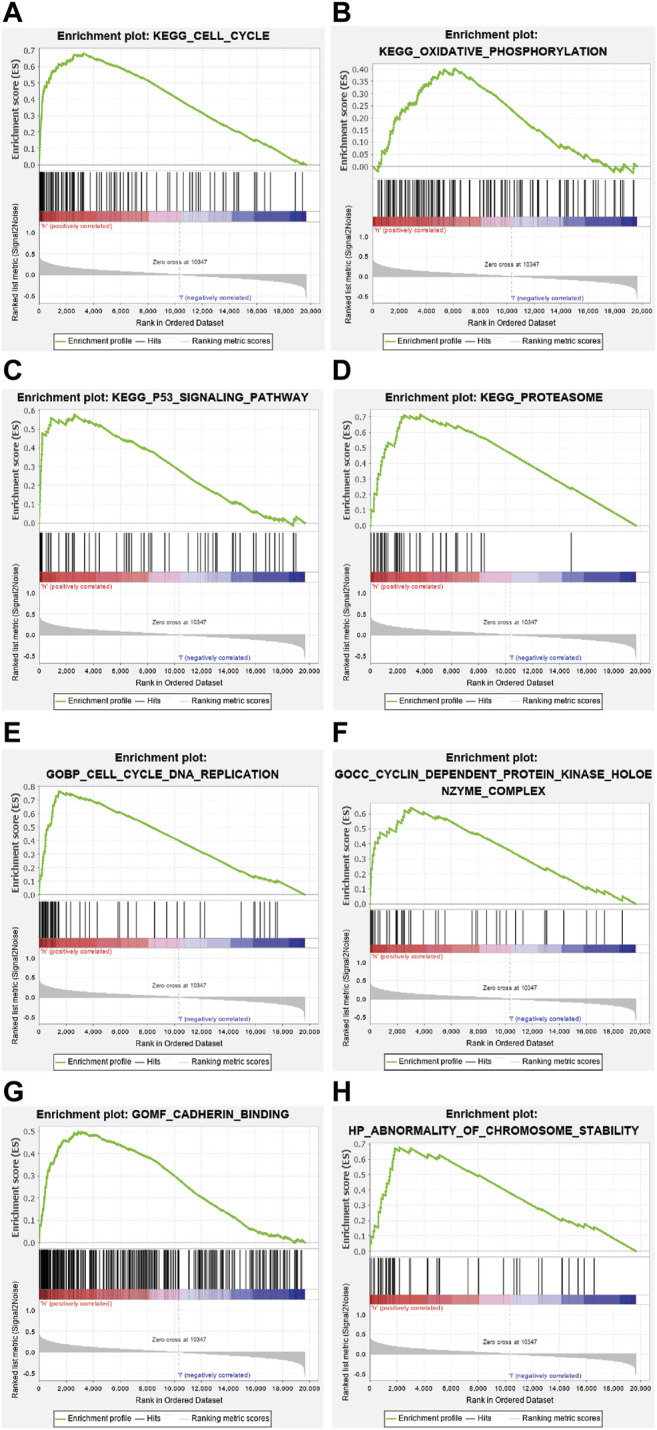
Kyoto Encyclopedia of Genes and Genomics (KEGG) enrichment analysis revealed that genes identified were involved in the following processes (**A**) cell cycle, (**B**) oxidative phosphorylation, (**C**) P53 signaling pathway, and (**D**) proteasome. Gene Ontology (GO) enrichment analysis demonstrated that the gene set was enriched in (**E**) DNA replication, (**F**) cell cycle regulation, (**G**) cell adhesion, and (**H**) chromosome variation.

The GO and KEGG enrichment analysis were performed on the DEGs of the above-mentioned high-and low-risk groups (Figure 6). The result of the analysis disclosed that the gene set was enriched in DNA replication, cell cycle regulation, cell adhesion, and chromosome mutation. As shown by the KEGG pathway enrichment analysis, screened genes were deeply involved in the cell cycle, oxidative phosphorylation, P53 signaling pathway, proteasomes, and so on. These results may provide a direction for researchers to study the mechanism of ferroptosis-related genes on lung adenocarcinoma in the future.

## Discussion

Lung adenocarcinoma is a common malignant tumor with a poor prognosis (Devarakonda et al., 2015). Predicting the outcome of tumors is of incontestable clinical significance in the diagnosis and treatment of patients with lung adenocarcinoma (Chen et al., 2021b). Previous studies have shown that chest CT, serum tumor markers, and TNM staging can be used as prognostic indicators of lung cancer (Calvayrac et al., 2017). However, there are certain limitations. In case of a risk of radiation exposure, the sensitivity and specificity are relatively low (Hoseok and Cho, 2015; Welch, 2017). Therefore, it is important to find predictors that can accurately predict the prognosis of patients with lung cancer.

According to new studies, ferroptosis has shown non-negligible potential in cancer treatment, especially for tumors that are not sensitive to traditional therapies (Liang et al., 2019; Proneth and Conrad, 2019). P53 is a widely studied gene that can suppress tumors, and inhibit the expression of cystine/glutamate antiporter at the transcriptional level to regulate the process of ferroptosis (Xie et al., 2017). In addition, studies have shown that the increase in iron-dependent reactive oxygen species can cause lipid peroxidation outside the mitochondria triggering ferroptosis, thereby inhibiting tumor development (Liang et al., 2019). In lung cancer, due to the upregulation of cystine/glutamate antiporter and the decrease in iron, ferroptosis is usually inhibited, which leads to the relapse and development of tumors (Lai et al., 2019). Therefore, our study aims to explore the relationshipbetween FRGs and the prognosis of patients with lung adenocarcinoma with the described underlying mechanism.

We obtained gene expression data with clinical data of lung adenocarcinoma from the public database. FRGs were extracted from the ferroptosis database. First, we identified seven target genes through DEGs and regression analysis. Multivariate Cox analysis was adapted to calculate regression coefficients and a prognostic model was developed, thereby dividing patients with lung adenocarcinoma into high- and low-risk groups. We observed that patients in the latter group lived longer OS than the other. Furthermore, we developed a nomogram according to the outcomes of multivariate Cox regression. ROC curve, calibration chart, and decision curve confirmed the prediction power of the nomogram. Compared with previous studies, the AUC value of the prognostic model based on ferroptosis-related genes (AUC = 0.823) was higher than that of the prognostic model based on metabolic genes (AUC = 0.767) (Yu et al., 2020), immune genes (AUC = 0.718) (Song et al., 2020), and autophagy genes (AUC = 0.810) (Wang et al., 2020).

A risk scoring model consisting of seven genes (TXNRD1, TRIB3, SLC2A1, CDKN2A, RRM2, SLC7A11, and G6PD) associated with ferroptosis was constructed. Thioredoxin reductase (TXNRD1) is overexpressed in lung cancer cells to maintain tumor survival, and this overexpression has been shown to be associated with clinical outcomes (Zhu et al., 2019). Studies have shown that TRIB3 is significantly upregulated in LUAD cell lines and tissues. TRIB3 gene knockdown significantly inhibited the growth and invasion of LUAD cells (Xing et al., 2020). The progression of lung adenocarcinoma can be inhibited by inhibiting SLC2A1 expression (Wang et al., 2017). CDKN2A is associated with DNA methylation and is closely related to the prognosis of patients (Tsou et al., 2007). Inhibition of RRM2 can activate STING signaling pathway and inhibit the enhancement of radiosensitivity of lung adenocarcinoma (Jiang et al., 2021b). Inhibition of SLC7A11 leads to poor prognosis in KRAS-mutated lung adenocarcinoma (Hu et al., 2020). Previous studies have shown that G6PD is an independent prognostic factor for lung adenocarcinoma (Nagashio et al., 2019).

Subsequently, we conducted a gene function enrichment analysis to reveal the mechanism of ferroptosis genes on lung adenocarcinoma. The results demonstrated that FRGs we screened were involved in DNA replication, cell cycle regulation, cell adhesion, chromosomal mutation, oxidative phosphorylation, P53 signaling pathway, and proteasome processes. Hence, FRGs can be used as predictors of lung adenocarcinoma prognosis and may play a crucial role in lung adenocarcinoma biology.

In conclusion, the study found seven ferroptosis-related genes by searching the database with prognostic value for patients with lung adenocarcinoma. We constructed a clinical prognostic model of FRGs, which possesses a good effect on predicting the survival rate of patients with lung adenocarcinoma, indicating that FRGs can very well predict the prognosis outcome of patients with lung adenocarcinoma, and play a crucial role in the relapse and development of lung adenocarcinoma.

## Data Availability

The original contributions presented in the study are included in the article/Supplementary Material, further inquiries can be directed to the corresponding author.

## Abbreviations

AUCs, areas under the curves; DEGs, differentially expressed genes; FDR, false discovery rate; FRGs, ferroptosis-related genes; GEO, Gene Expression Omnibus; GO, Gene ontology; KEGG, Kyoto Encyclopedia of Genes and Genomics; NSCLC, non-small cell lung cancer; OS, overall survival; ROC, receiver operating characteristic; TCGA, The Cancer Genome Atlas Program
