# Supplementary material for: Construction of a Prognostic Model in Lung Adenocarcinoma Based on Ferroptosis-Related Genes
Source: Front Genet. 2021 Sep 22;12:739520. doi: 10.3389/fgene.2021.739520 (PMC8493116; doi:10.3389/fgene.2021.739520)
Supplement: Supplementary file 4 [file Image1.pdf]

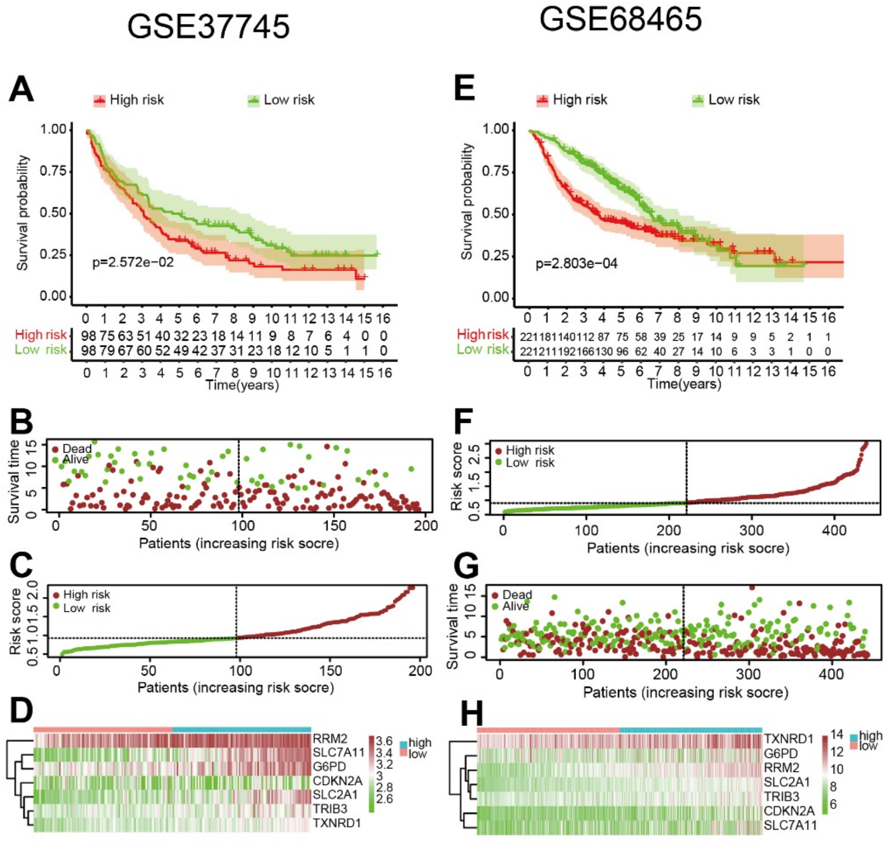


Supplementary material Figure 1 (A, E) Kaplan-Meier survival analysis of patients with lung adenocarcinoma in high-and low-risk groups; (B, F) The overall survival rate and status of patients with lung adenocarcinoma; (C, G) The distribution of risk scores; (D, H) The expression level of these 7 ferroptosis-related genes in the low-risk group and the high-risk group, the cool color represents low expression, while the warm color represents high expression.
